# Magnetic Ring Multi-Defect Stereo Detection System Based on Multi-Camera Vision Technology †

**DOI:** 10.3390/s20020392

**Published:** 2020-01-10

**Authors:** Xinman Zhang, Weiyong Gong, Xuebin Xu

**Affiliations:** 1 School of Electronics and Information Engineering MOE Key Lab for Intelligent Networks and Network Security, Xi’an Jiaotong University, Xi’an 710049, China; zhangxinman@mail.xjtu.edu.cn; 2 Guangdong Xi’an Jiaotong University Academy, Foshan 528000, China; ccp9999@126.com

**Keywords:** multi-camera vision technology, magnetic ring defect detection, stereoscopic inspection, image processing

## Abstract

Magnetic rings are the most widely used magnetic material product in industry. The existing manual defect detection method for magnetic rings has high cost, low efficiency and low precision. To address this issue, a magnetic ring multi-defect stereo detection system based on multi-camera vision technology is developed to complete the automatic inspection of magnetic rings. The system can detect surface defects and measure ring height simultaneously. Two image processing algorithms are proposed, namely, the image edge removal algorithm (IERA) and magnetic ring location algorithm (MRLA), separately. On the basis of these two algorithms, connected domain filtering methods for crack, fiber and large-area defects are established to complete defect inspection. This system achieves a recognition rate of 100% for defects such as crack, adhesion, hanger adhesion and pitting. Furthermore, the recognition rate for fiber and foreign matter defects attains 92.5% and 91.5%, respectively. The detection speed exceeds 120 magnetic rings per minutes, and the precision is within 0.05 mm. Both precision and speed meet the requirements of real-time quality inspection in actual production.

## 1. Introduction

Magnetic materials are commonly used in electronic circuits as anti-interference components. As the main product, magnetic rings are widely used in aerospace, military electronics, automobiles, sensors, the petrochemical industry, magnetic drives and other fields [1]. During the production process, due to the production environment and processing procedures, defects, such as crack, adhesion, pitting, foreign matter and fiber defects, will appear on the surface of the magnetic ring. These defects would affect the appearance and performance of industrial products. Therefore, high-efficiency and high-precision surface detection of magnetic rings is crucial for manufacturing enterprises. However, surface defect detection and height measurement of magnetic rings by manual inspection, which is currently widely used, are costly, inefficient, low-speed and low-accuracy. Hence, it is significant to design an automated system to complete real-time defect detection and height measurement for magnetic rings.

In recent years, many excellent technical solutions for defect detection have been proposed by researchers. The ultrasonic testing technique [2,3], eddy current testing technique [4,5,6] and vision inspection technique [7,8,9,10] are widely used. Among them, in practical production, the vision inspection technique has been successfully adopted for surface detection of industrial products because of its convenience, effectiveness and cost performance. Li and Ren [11] developed an intelligent vision detection system for discrete rail surface defects. Tsai and Hsieh [12] proposed a fast image alignment method by using the expectation-maximization (E-M) technique, which accomplished the positioning and defect inspection of printed circuit boards (PCBs). Furthermore, an entropy-rate clustering algorithm combined with prior shape constraint was proposed by Chen [13] to detect defects and deformations across an imaged can-end surface. Ko and Rheem [14] inspected the surface defects of solar wafers by using the local binarization method.

Furthermore, aimed at the inspection of magnetic ring surfaces, researchers have carried out active research. By using the ultrasonic testing technique, Xu [15] designed a programmable filter based on the MAX262 chip, and used the fourth-order Chebyshev bandpass filter to extract weak vibration signals for detecting cracks in the magnetic ring. However, the detection of defects other than cracks was not considered. Using the vision inspection technique, Yu [16] designed an automatic vision-based detection system and used an improved BHPF (butterworth high pass filter) filter for denoising. Nevertheless, the designed system only completed the detection of the magnetic ring end face, which is not comprehensive. Furthermore, Li and Zhang [17] proposed a surface defect extraction method for magnetic rings based on the adaptive Canny algorithm of the edge-detection method and masking technology. However, the detection of the inner torus was not considered.

To measure the multiple dimensions of magnetic rings quickly and to detect and classify the defects precisely, this paper develops a magnetic ring intelligent detection system based on multi-camera vision technology. A wide range of aspects is considered to ensure that no defect or surface is missing, making our system more comprehensive than the systems mentioned above. Trapped by multiple tasks and diverse defects, the traditional single vision system cannot obtain enough information. So, multiple vision machines are used in the system, which guarantees high-speed defect detection on multiple surfaces of three-dimensional magnetic rings. Two image processing algorithms are proposed, namely, the image edge removal algorithm (IERA) and magnetic ring location algorithm (MRLA), respectively, which effectively solve the defect segmentation problem. Finally, by using reasonable thresholds obtained through experiments, connected domain filtering methods are indicated to localize defects quickly and accurately.

The rest of this paper is organized as follows. Section 2 describes the hardware and software components of the multi-vision machine intelligent detection system; Section 3 describes the defect detection algorithm for each surface and the height measurement method; Section 4 demonstrates the detection results of the system. Finally, the conclusions are given in Section 5.

## 2. System Design

### 2.1. Hardware Design

**Figure 1:**
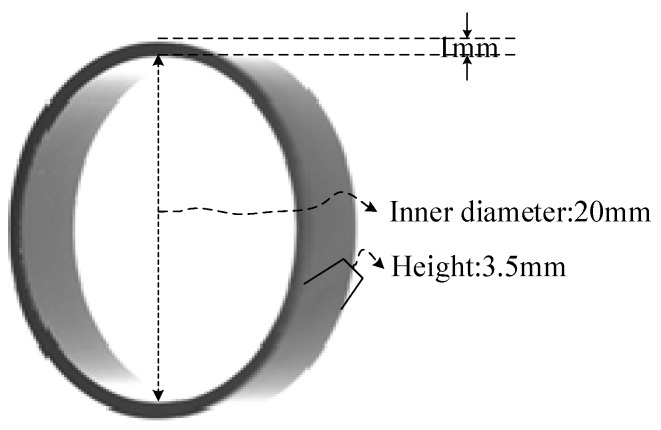
Physical image of the magnetic ring.

As can be seen from Figure 1, the outer diameter and inner diameter of the magnetic ring to be tested are 22 and 20 mm. The height is 3.5 mm, and the color is gray. The structure is complicated and the types of defects are various, so how to realize the real-time online detection of all defects and surfaces is a difficult problem. One approach is using a single vision machine and rotational shooting mode, but this demands a high-precision rotary positioning system and the detect speed is too low to meet the requirements. Considering the fact that defects are distributed on all sides of the magnetic ring, we propose a solution that can complete image acquisition and processing synchronously by using multiple high-speed vision machines.

**Figure 2:**
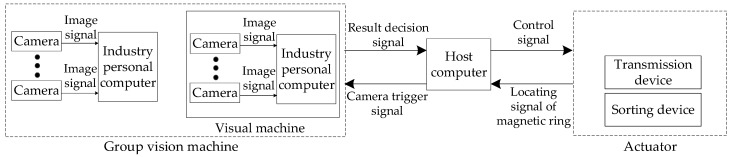
Structural diagram of the magnetic ring detection system.

This system mainly includes three modules, which are the visual inspection module, actuator module and host computer module (as shown in Figure 2). The visual inspection module contains six vision machines and achieves multi-view image collection, image processing and test results sending. The vision machine integrates camera control, card processing and serial communication to solve industrial automation production problems [18]. The host computer takes charge of the judgment results summary of all the vision machines and the control of the executing mechanism. The actuator module, consisting of a feeding device, a transmission device and a sorting device, completes the mechanical operation of the magnetic ring. The feeding device is responsible for feeding magnetic rings into the transmission device at a certain speed. The transmission device is in charge of transmitting magnetic rings from the feeding device to the sorting device. The sorting device finishes final defect classification according to the control signal of the host computer.

Relying on the elaborate design, the actuator could successfully realize non-destructive detection. The vibrating feeding plate is used as the feeding device, whose vibration frequency is adjustable to ensure uniform feeding speed and avoid secondary damage. Meanwhile, since both the upper and lower end faces need to be detected, a glass turntable is employed as the driving device. In this way, by setting cameras and light sources above or below the glass disk, images are taken without flipping the magnetic ring. Furthermore, a solenoid valve is used as the sorting device to ensure the response speed and mechanical force. Ultimately, there will be no secondary damage to the magnetic ring.

Besides, the hardware of the system has high expansibility and can be adjusted according to the actual requirements. Benefitting from multiple vision machines, this system has a high processing speed and a wide range of perspectives, which can deal with three-dimensional part detection problems. Nevertheless, with the use of multiple vision machines, the cost of the system is not that inexpensive. Furthermore, there are many components in the hardware system, so the complexity of the device brings some difficulties to the later management.

**Figure 3:**
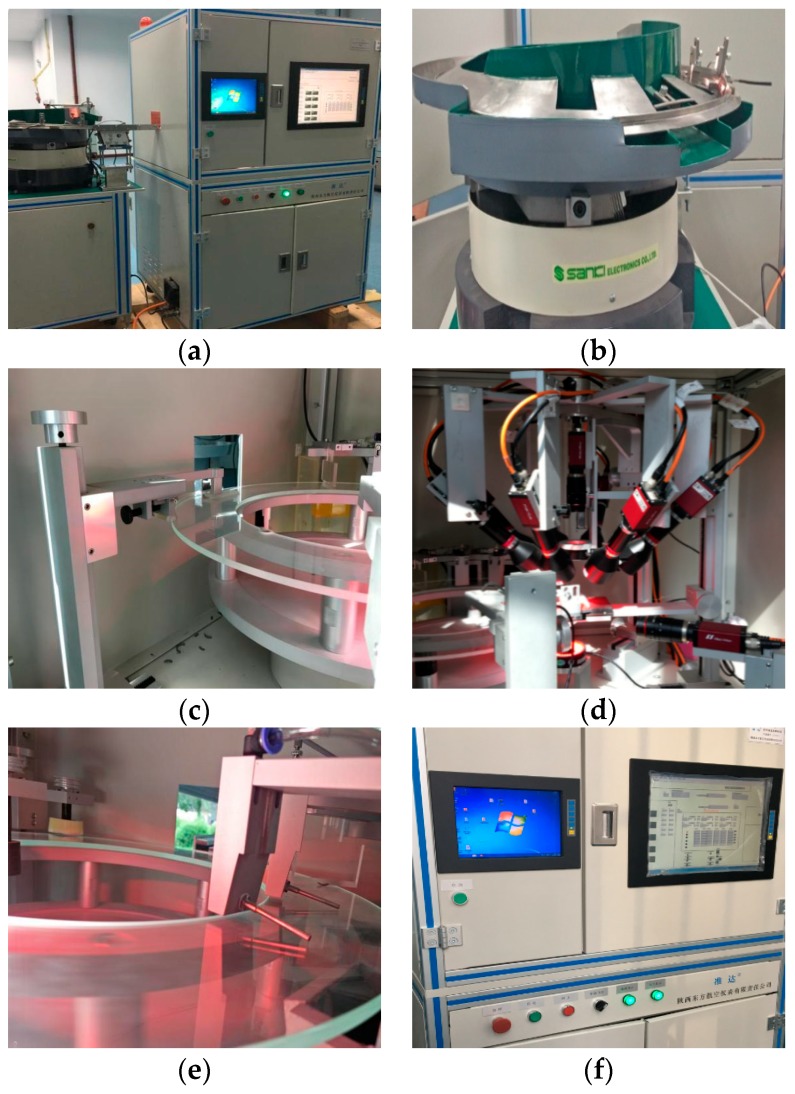
The prototype of the magnetic ring detection system: (**a**) system appearance; (**b**) feeding device; (**c**) precision turntable and slide rail limit device; (**d**) group vision machine and light source; (**e**) sorting device; (**f**) host computer and console.

**Figure 4:**
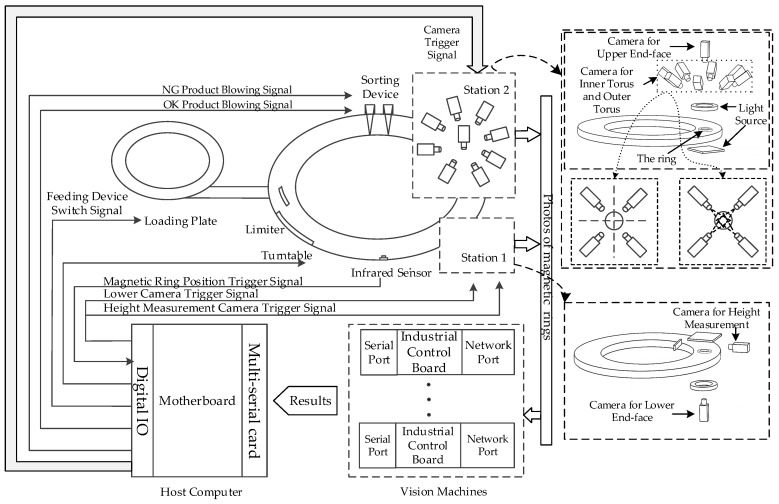
Total plan of the magnetic ring defect detection system.

Figure 3 shows the prototype of this multi-vision magnetic ring quality inspection system. To further illustrate the hardware system, a simplified diagram of the entire system is drawn, as shown in Figure 4. First, magnetic rings are fed into the system through the vibrating feeding plate. Then, it reaches the camera shooting point after adjusting the position and completing the counting. Subsequently, this system uses two stations to photograph the magnetic ring and detect defects. Station 1 contains a binocular vision machine and two cameras. These two cameras are responsible for measuring the height and detecting the lower end face. Station 2 consists of five vision machines with a total of nine cameras. Due to the inner and outer toruses of the magnetic ring being curved surfaces, the view of a single camera cannot cover all toruses. So, among them, four cameras are responsible for taking pictures of the outer surface, and four cameras are responsible for taking pictures of the inner surface. The last camera with a vertical view is located on the platform to detect the upper end face. After taking the picture, vision machines receive the image and perform defect detection. Then, all detection results are sent to the host computer. Finally, when the magnetic ring reaches the sorting device, the qualified and unqualified magnetic rings are separated. 

### 2.2. Software Design

Based on the system hardware, the software of the proposed system is developed on the MFC and OpenCV platforms. If any defect is found, the software will mark and output the defect’s height measurement results. It includes four modules, which are the camera control module, interactive interface module, magnetic ring defect detection module, and detection result communication and storage module. The camera control module mainly performs tasks such as camera control, camera status feedback, camera parameter setting, camera mode selection, image storage buffering and release. The interactive interface module is in charge of the interaction between the user and the system, including the camera control area, real-time image display area, result display area, threshold selection area and magnetic ring-type selection area. The defect detection module integrates all the methods proposed in the paper and takes charge of real-time detection and marking of images. The detection result communication and storage module adopts the serial communication mode and completes the transmission task of the defect detection result from the vision machine to the upper computer.

**Figure 5:**
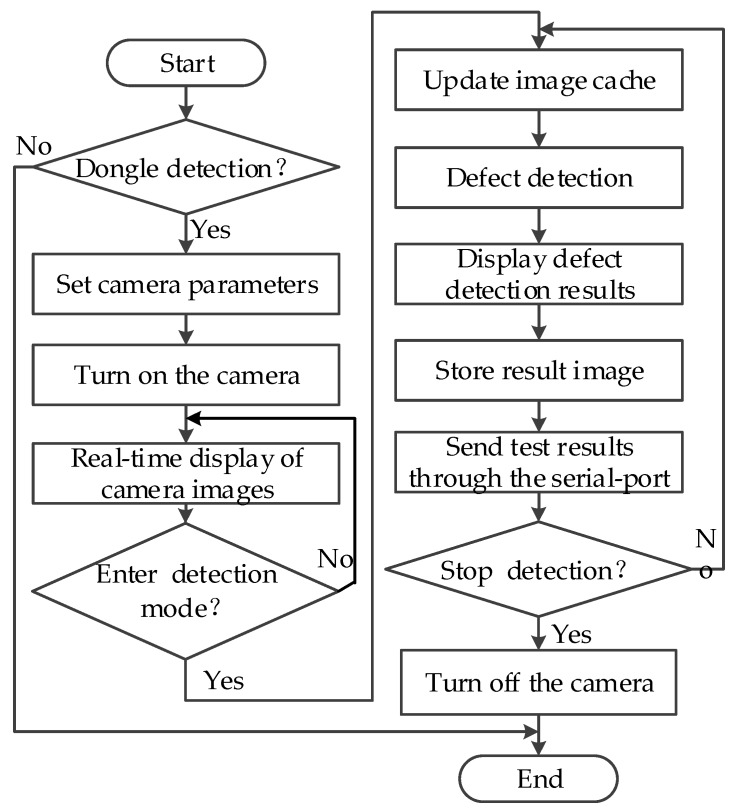
Flowchart of the software system.

**Figure 6:**
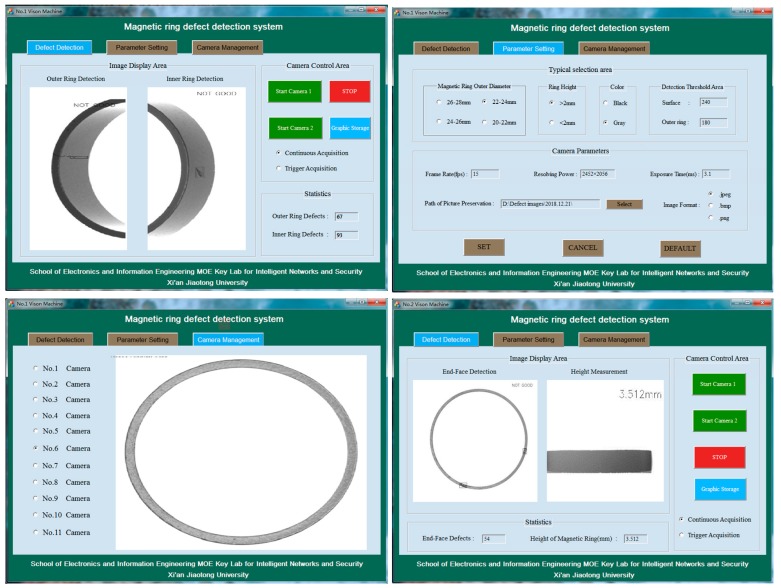
Magnetic ring detection program interface for No. 1 vision machine and No. 2 vision machine.

The software runs on six visual machines. The interactive interface and defect detection modules of different visual machines are adjusted according to the installation position of the camera, the number of cameras and the characteristics of the magnetic ring image. However, the software framework and operating procedures are the same for each machine. The flow chart of software operation is shown in Figure 5, and the interactive interface can be seen in Figure 6.

## 3. Methods

This section presents the details of the proposed approach for magnetic ring defect detection. The proposed methods include image preprocessing, IERA, MRLA, connected domain filtering method and the final part to measure the height of magnetic rings. The preprocessing step segments the suspected defect area with IERA and MRLA. Eventually, connected domain filtering identifies defects by analyzing morphological features. The details are as follows.

### 3.1. Image Preprocessing

The defects to be detected on the surface include cracks, pits, adhesions, foreign matters, fibers, etc. Aimed at simplifying the detection problem, all defects are categorized into three categories, which are crack, fiber and large-area defects. Meanwhile, due to inherent problems with the system hardware, positioning error and uneven illumination will occur in magnetic ring images.

**Figure 7:**
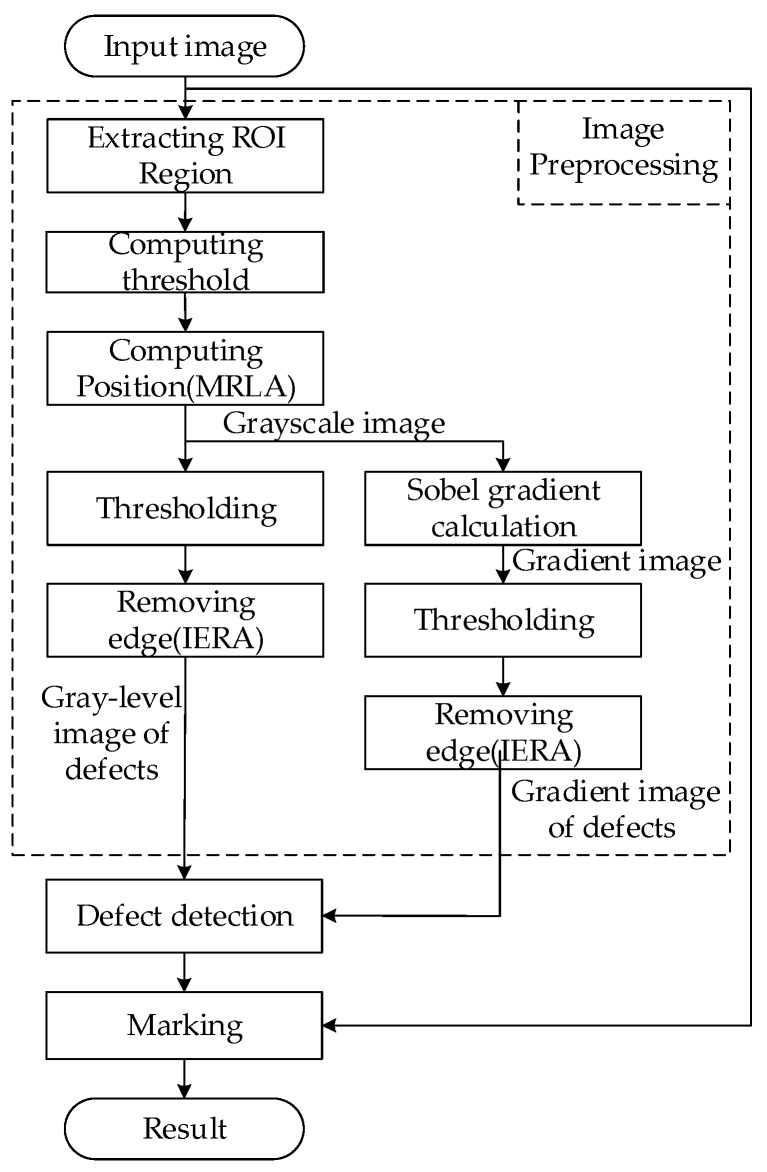
Flow diagram of the image processing.

To eliminate the unnecessary information and extract the key features in the image, a series of image preprocessing steps is performed. Figure 7 depicts the process of image processing. It is noticed that the gray-level and gradient defect features should be extracted separately in the preprocessing stage as the basis. Firstly, after extracting the ROI (region of interest) region of the input image, Otsu’s [19] method is used to compute the gray threshold. Then, MRLA is used to calculate the position of the magnetic ring, solving the problem of positioning error. Subsequently, on the one hand, we perform sub-region gray threshold segmentation [20] to solve the uneven illumination problem and use IERA to acquire gray-level defect images. On the other hand, the Sobel operator is used to perform the gradient calculation. Finally, IERA is utilized to obtain the gradient image of defects.

**Figure 8:**
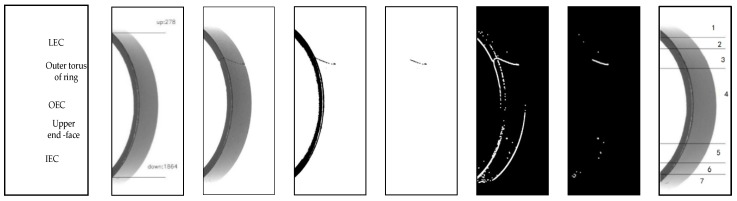

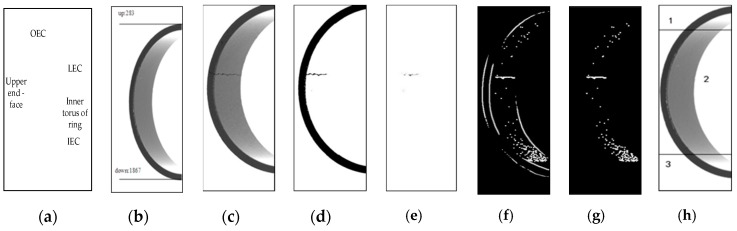
Torus image processing: (**a**) annotation of torus images; (**b**) location result; (**c**) original defect image of torus (2056 × 600); (**d**) torus gray binary image; (**e**) edge-removed torus gray binary image; (**f**) torus gradient binary image; (**g**) edge-removed torus gradient binary image; (**h**) segmentation of torus images.

The magnetic ring appears as a curved surface in the image. The middle area of images is dark and the areas at both ends are bright due to the designed lighting system and the location of the camera (Figure 8h). To solve this uneven illumination problem, as mentioned above, sub-region gray threshold segmentation is performed. First, Otsu’s method computes an optimal threshold that minimizes the intra-class variance. Suppose $p_{i}$ is the percentage of pixels with value i. The threshold T is computed according to Equation (1).

(1) $$\underset{T}{\min}\sigma^{2}{}_{\mathrm{int}ra}(T)=n_{B}(T)\sigma_{B}^{2}(T)+n_{A}(T)\sigma_{A}^{2}(T)$$

where

(2) $$n_{B}(T)=\sum_{i=1}^{T-1}p_{i},\;\mathrm{and}\;\sigma_{B}^{2}(T)=\mathrm{variance}\;\mathrm{of}\;\mathrm{pixels}\;\mathrm{below}\;T.$$

(3) $$n_{A}(T)=\sum_{i=1}^{L-1}p_{i},\;\mathrm{and}\;\sigma_{A}^{2}(T)=\mathrm{variance}\;\mathrm{of}\;\mathrm{pixels}\;\mathrm{above}\;T.$$

According to the illumination performance, the target magnetic ring in the image is divided into i regions. The threshold value $T_{i}$ of the i−th region is defined by Formula (4).

(4) $$T_{i}=T-D_{i}$$

**Figure 9:**
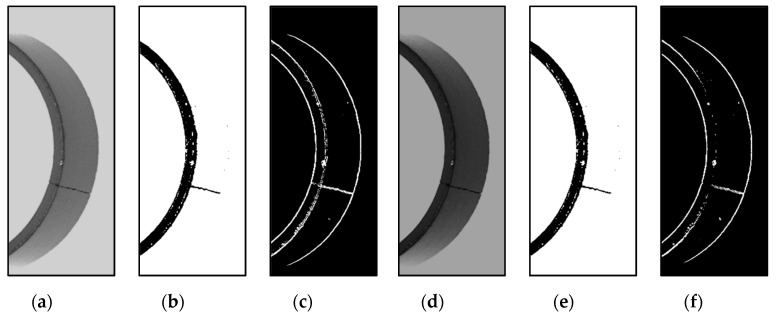
(**a**–**f**) Torus gray binary images.

The threshold decay term $D_{i}$ is obtained through experiments. Suppose H is a grayscale image and G stands for the image after thresholding. The i−th region is binarized using Formula (5). An example is shown in Figure 8d. To prove the stability of the used image preprocessing method in relation to illumination, we change the illumination conditions (see Figure 9a,d) and conduct experiments with all other conditions being equal. The results confirm that the method has good robustness to illumination.

(5) $$G_{i}(m,n)=\{\begin{array}{ll} 1, & if\;H_{i}(m,n)>T_{i}\\ 0, & otherwise \end{array}$$

### 3.2. Magnetic Ring Location Algorithm

For the sake of carrying out accurate inspection, the position of the magnetic ring should be determined quickly. On account of the hardware, the position of the magnetic ring is unfixed in the system and the location migration occurs mainly in one direction. To solve the problem, MRLA (see Algorithm 1) is proposed to calculate the position of the magnetic ring in the image. Figure 8b shows examples of torus image location results.

**Figure 10:**
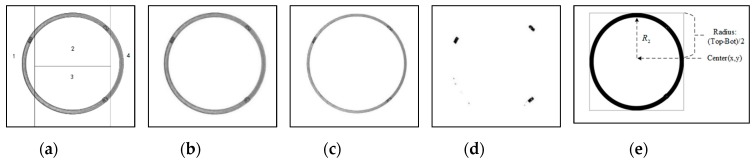
End face image processing: (**a**) segmentation of end face images; (**b**) end face image (2452 × 2056); (**c**) edge-removed end face image; (**d**) edge-removed end face binary image; (**e**) location result and annotation.

As for end face images, Algorithm 1 is adopted to acquire the top position top, and the bottom position bot. Suppose $T_{l}$ is the positioning threshold. Then, in step 5, the scanning direction is changed to left-to-right. Finally, the left position left and the right position right are obtained. Let Center(x,y) denote the magnetic ring center position, whereas x=(left+right), and y=(top+bot)/2. An example is shown in Figure 10e.
**Algorithm 1** Magnetic Ring Location Algorithm1: Given a grayscale torus image $H\in R^{M\times N}$.2:  Use sub-region gray threshold segmentation to obtain a binary image $G_{h}$.3:  **for** I = 0, …, M **do**4:     Scan $G_{h}$ from top to bottom.5:     Count the number δ of black dots.6:     **if**
$\delta >T_{l}$
7:         Get the up position up=i. 8:     **end**9:  **end**10: **for** I = 0, …, M **do**11:   Scan $G_{h}$ from bottom to top.12:   Count the number δ of black dots.13:   **if**
$\delta >T_{l}$
14:     Get the down position down=i. 15:   **end**16:  **end**17: Output the location result up and down.

### 3.3. Image Edge Removal Algorithm

In the image preprocessing procedure, it is found that gradient binary images and grayscale images contain the inner edge contour (IEC) of the upper end face, the outer edge contour (OEC) of the upper end face, and the lower edge contour (LEC) of the magnetic ring, as shown in Figure 8a. These edges will interfere with the subsequent defect filtering. To better isolate defects from images, IERA is proposed to eliminate these edges.

**Figure 11:**
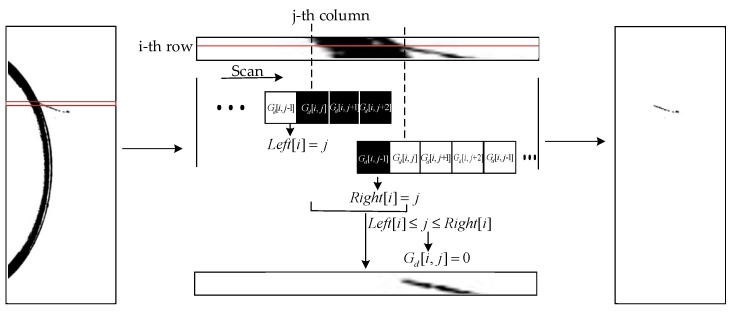
Sketch of the image edge removal algorithm.

The edge of the magnetic ring behaves differently in different kinds of pictures. To extract the defect features of different surfaces, IERA is divided into three parts. Algorithm 2 Part I, Part II and Part III describe IERA for torus grayscale images, torus gradient images and end face binary images, respectively. Figure 8c–g shows the process of IERA. By means of Algorithm 2 Part I, torus gray binary images after removing the upper end face are shown in Figure 8e. To illustrate the algorithm, a sketch is shown in Figure 11.

Sometimes, defects, such as recessive cracks and fibers, are inconspicuous in gray binary images. However, they could be easily observed in gradient binary images. By using Algorithm 2 Part II, the edge-removed torus gradient image can be obtained (see Figure 8g). Besides, according to the imaging characteristics of the end face of magnetic rings, through Algorithm 2 Part III, we can obtain the edge-removed end face image (see Figure 10c). Furthermore, after sub-region gray threshold segmentation, an edge-removed end face binary image with defect features is shown in Figure 10d.

**Algorithm 2** Image Edge Removal Algorithm Part I1:  Given a grayscale image $H\in R^{M\times N}$.2:  Process and get gray binary image $G_{d}$.3:  **for** I = 0, …, M **do**4:  Set the array of IEC to Left[i], set the array of OEC to Right[i].5:  **for** j = 0, …, N − 2 **do**6:   **if**
$G_{d}[i,j+k]==0(k=0,1,2)$
7:       Get Left[i]=j.8:   **end**9:   **end**10:     **for** j = Left[i], …, N − 4 **do**11:   **if**
$G_{d}[i,j+k]==255(k=0,\ldots ,4)$
12:       Get Right[i]=j.13:   **end**14:  **end**15:  **for** j = 0, …, N **do**16:     **if**
Left[i]≤j≤Right[i]
17:       Set $G_{d}[i,j]=255$
18:     **end**
19:   **end**20: **end**21: Output the edge-removed gray binary image $G_{d}$.Part II1:Given a grayscale image $H\in R^{M\times N}$.2:Obtain a binary image $G_{d1}$ and an image $G_{h}$ with high threshold binarization. Use Sobel operator to obtain gradient binary image $G_{w}$.3:**for** i = 0, …, M **do**4:    Set the array of IEC to TopL[i] , set the array of LEC Bot[i] and set the array of OEC to Top[i].5:    **for** j = 0, …, N − 4 **do**7:     **if**
$G_{h}[i,j+k]==0\;(k=0,\cdots ,4)$
8:           Get TopL[i]=j.9:     **end**10:    **end**11:    **for** j = TopL[i] , …, N − 4 **do**13:     **if**
$G_{h}[i,j+k]==255\;(k=0,\cdots ,4)$
14:           Get Bot[i]=j. 15:     **if**
$G_{d1}[i,j+k]==255\;(k=0,\cdots ,4)$
16:           Get Top[i]=j.17:     **end**18:    **end**19:    **for** j = 0, …, N **do**20:     **if**
0≤j≤Top[i]+15 or |j−Bot[i]|≤20
21:           Set $G_{w}[i,j]=0$.22:     **end**23:    **end**24:
**end**
25:Output the edge-removed torus gradient image $G_{w}$.Part III1:Given a grayscale end face image $H\in R^{M\times N}$.2:Process and get binary image $G_{h}$.3:Create a white circle $C_{1}$ with $\left(Center\left(x,y\right),\left(Top+Bot\right),w_{1}\right)$ as its center, radius and line width, respectively.4:**for***j* = *y*, *y* − 1, …,4 **do**5:  Scan upward from Center(x,y).6:  **if**
$G_{h}[x,j-k]==0\;(k=0,\cdots ,4)$
7:         Get $R_{2}=y-j$.8:  **end**9: **end**10:Create a white circle $C_{2}$ with $\left(Center\left(x,y\right),R_{2},w_{1}\right)$ as its center, radius and line width, respectively.11:Use $C_{1}$ and $C_{2}$ to remove edges.12:Output the edge-removed grayscale end face image.

### 3.4. Connected Domain Filtering Method

After preprocessing, the remaining connected components represent the potential defect areas. As the size and shape of defect should meet certain conditions to be mechanically significant, the connected components are further filtered by thresholds [21]. In accordance with statistics, the specific characteristics of the three types of defects are as follows:

- Crack defect: It can be categorized into obvious crack and recessive crack. The obvious crack is shown as a thick line in the image and has a strong contrast with the torus region. The recessive crack is opposite to the obvious crack.
- Large-area defect: It is characterized by obvious protrusion or depression, a strong contrast with the surrounding area and a large defect area.
- Fiber defect: It has the characteristics of low gray-level discrimination with the surrounding area, inconspicuous protrusion, irregular shape, and fine lines.

For obvious cracks in contact with the end face of the magnetic ring, detection is performed by scanning the upper edge array Top[i] to detect the curvature transition point [22]. Let $T_{q}$ denote the curvature threshold. If Formula (6) is satisfied, a crack defect is found.

(6) $$Top[i][j]-Top[i-8][j]>T_{q}(i=up+8,up+9,\ldots ,down;j=1,2,\ldots ,7)$$

Large-area defects and the rest of the obvious cracks have common characteristics, such as large contrast with normal torus, obvious morphological features in binary images, and large area of connected domains. So, same method based on connected domain filtering is used to solve the two similar problems (see Algorithm 3 Part I below). Let us define the minimum bounding rectangle T (with length H and width W) of the connected domain. Let $T_{\mathrm{wh}}$ denote the length and width ratio threshold of the connected domains and $R_{\mathrm{wh}}$ denote the ratio of the connected domain to the area of its minimum bounding rectangle. $T_{h}$ and $T_{w}$ represent the length threshold and the width threshold of T, respectively. Finally, suppose the threshold of the area of connected domains is denoted by $T_{s}$.

**Algorithm 3** Connected Domain Filtering MethodPart I 1:Give a gray binary image $G_{d}$ or a gradient binary image $G_{w}$.2:Process and get $G_{d}$’s anti-color image $G_{dc}$.3:Get the connected domain set Q and the number k of the connected domain.4:**for***i* = *1*, …, *k*
**do**5:  Get the area $S_{i}$ of the connected domain $Q_{i}\in Q$, the minimum bounding rectangle $T_{i}$ (with length $H_{i}$ and width $W_{i}$).6:  Set each value of $\left(T_{\mathrm{wh}},R_{\mathrm{wh}},T_{h},T_{w},T_{s}\right)$.7:  **if**
condition is satisfied8:     $Q_{i}$ is judged to be a defect.9:  **end**10:
**end**
11:Output the results.Part II1:Given a gradient binary image $G_{w}$.2:Get the width W of $G_{w}$.3:Set the step length step and a rectangular search box Sbox with a width of $w_{b}$ and a length of $h_{b}$. Set number of connected domains $N_{i}=0$.4:**for***i* = 1, …$w_{b}+i*step$, …, W do5: Use Sbox to traverse $G_{w}$ from top to bottom in steps of step.6: For the i−th
Sbox, get k connected domains.7: **for**
*j* = 1, …, *k*
**do**8:  Get the area $S_{j}$ of the connected domain $Q_{j}\in Q$, the minimum bounding rectangle $T_{j}$ (with length $H_{i}$ and width $W_{j}$).9:  Set each value of $\left(T_{\mathrm{wh}},R_{\mathrm{wh}},T_{h},T_{w},T_{hs},T_{ss}\right)$.10:  **if**
condition1 is satisfied.11:     $N_{i}+=1$ and save the coordinate of the connected domain $\left(x_{j},y_{j}\right)$.12:  **end**13:  **end**14:  Set $T_{n}$ and $T_{hs}$.15:  **if**
condition2 is satisfied.16:  The i−th
Sbox contains defects.17:  **end**18:
**end**
19:Output the results.

**Figure 12:**
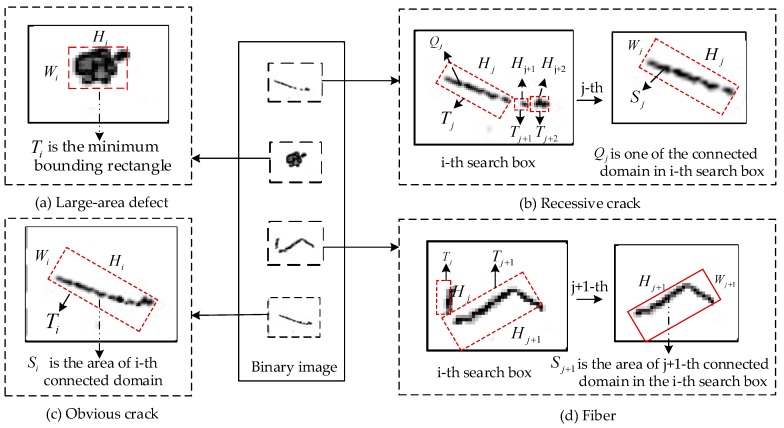
Illustratory diagram of connected domain filtering.

With regard to obvious cracks, the connected domain is represented as a thick straight line (see Figure 12c). For the connected domain and its minimum bounding rectangle, by extensive experiments, we define that if conditions, such as $H>T_{h}$, $W<T_{w}$, $H/W>T_{wh}$ and $S/(W\times H)>R_{wh}$, are satisfied, connected domains are identified as obvious cracks. So, the filter criterion condition can be expressed as (7).

(7) $$H_{i}/W_{i}>T_{wh}\&\&S_{i}/(W_{i}\times H_{i})>R_{wh}\&\&H_{i}>T_{h}\&\&W_{i}<T_{w}$$

For large-area defects, connected domains are characterized by large, irregular shapes (see Figure 12a). On the basis of statistics, it is defined that if conditions, such as $H>T_{h}$, $W>T_{w}$, $H/W>T_{wh}$ and $S/(W\times H)>R_{wh}$, are satisfied, connected domains can be defined as large-area cracks. So, the filter criterion condition can be expressed as (8).

(8) $$S_{i}>T_{s}\&\&S_{i}/(W_{i}\times H_{i})>R_{wh}\&\&W_{i}>T_{w}\&\&H_{i}>T_{h}$$

Fiber defects and recessive crack defects do not have strong contrast with normal torus. In binary images, they appear as several small connected domains distributed in a certain region. Therefore, these two defects are filtered according to the characteristics of connected domains and the relationship between connected domains. Let $T_{n}$ denote the number threshold of the connected domain in the search box. $T_{hs}$ represents the width sum threshold of the connected domain, and $T_{ss}$ delegates the area sum threshold of the connected domain.

Recessive cracks appear (Figure 12b) as several disconnected lines in the processed binary image. For example, Figure 12b describes a recessive crack and its made up of three line segments. Like the obvious crack, in Algorithm 3 Part **II**, the filter criterion condition1 is expressed as condition (9) to identify whether the segments are part of a crack defect. Furthermore, what really matters is the relationship between the segments. $\sum H_{j}$, the sum of the minimum bounding rectangle length of adjacent connected domains in a search box Sbox, is selected as the filtering criterion. Through extensive experiments, we define that if conditions $N_{i}>=T_{n}$ and $\sum H_{j}\geq T_{hs}$ are satisfied, Sbox is determined to contain a recessive crack. So, replacing condition2 with condition (10), the method can detect the recessive crack correctly.

(9) $$H_{j}/W_{j}>T_{wh}\&\&S_{j}/(W_{j}\times H_{j})>R_{wh}\&\&H_{j}>T_{h}\&\&W_{j}<T_{w}$$

(10) $$N_{i}>=T_{n}\&\&\sum_{j=1}^{N_{i}}H_{j}\geq T_{hs}$$

Fiber defects appear as several irregularly disconnected connected domains in the processed binary image (see Figure 12d). In the first place, condition (11) is introduced to take the place of condition1 to identify whether connected domain $Q_{j}$ is part of a fiber. Subsequently, by extensive experiments, it is found that the sum $\sum S_{j}$ of the connected domain area is more useful in defect detection than the sum $\sum H_{j}$ of minimum bounding rectangle length. Thus, the filter criterion condition2 is expressed as (12).

(11) $$S_{j}>T_{s}\&\&W_{j}>T_{w}\&\&H_{j}>T_{h}$$

(12) $$N_{i}>=T_{n2}\&\&\sum_{j=1}^{N_{i}}S_{j}\geq T_{ss}$$

### 3.5. Measurement Method of Magnetic Ring Height

**Figure 13:**
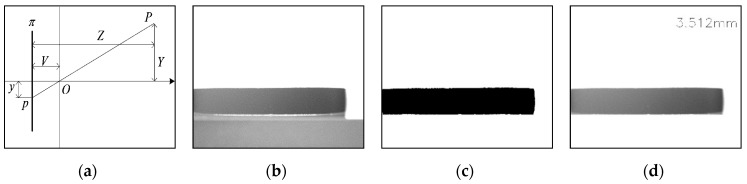
(**a**) Schematic diagram of spatial position measurement in the vertical direction; (**b**) a magnetic ring height image; (**c**) a binary image after removing the glass area; (**d**) a result of height measurement.

In addition to defect detection, height measurement is also an important target of this system. In the magnetic ring height image, the glass wheel’s area is fixed (see Figure 13b) and can be removed by setting the fixed area of the grayscale image to white (see Figure 13c). Then, MRLA is used to calculate the position of the magnetic ring and get the upper position up and lower position down. Subsequently, the pixel height of the magnetic ring height=down−up is obtained.

According to the imaging principle of the camera, the spatial position measurement diagram of the target vertical direction in the image is shown in Figure 13a. Let π denote the imaging plane and O denote the lens center of the camera. V presents the image distance from the light center to the image plane, and Z defines the distance from the object to the image plane. From the triangle similarity principle, we can obtain:

(13) $$Y=\frac{Z-V}{V}y$$

Formula (Z−V)/V is fitted through experiments, and y=height is substituted into Formula (13) to obtain the actual height of the magnetic ring. Through practical verification, ultimately, the precision of height measurement is about 0.02 mm.

## 4. Experiments

### 4.1. Examples of Defect Detection

All the proposed algorithms and methods are applied to the magnetic ring detection system. Then, we perform some experiments to check the operation of the system. According to the manufacturing process, we find that crack and fiber defects mainly appear in the inner and outer ring surfaces. Hence, we can detect them from the inner and outer ring surfaces. It is noteworthy that various kinds of large-area defects will appear on the outer ring and the end face, and the large-area defect on the inner ring is an adhesion defect.

**Figure 14:**
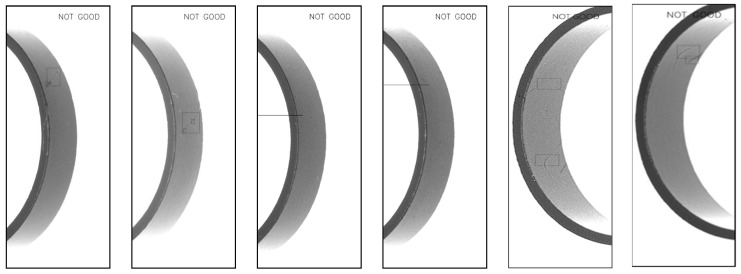
Detection results of fiber defects.

**Figure 15:**
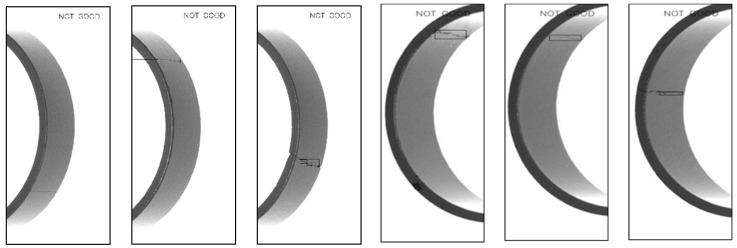
Detection results of crack defects.

**Figure 16:**
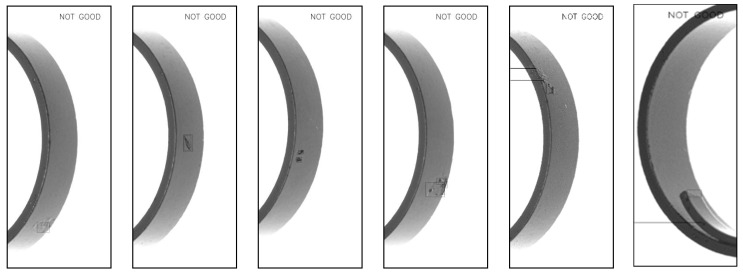

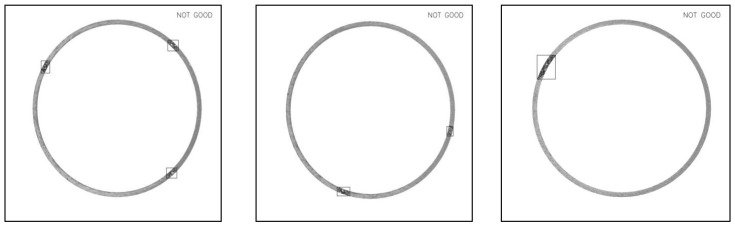
Detection results of large-area defects.

The results are shown below, including magnetic ring height (Figure 13d), fiber defects (Figure 14), crack defects (Figure 15) and large-area defects (Figure 16). For different defects located on different surfaces, the system can accurately judge whether it is a defect and mark the position.

### 4.2. Statistical Results of Defect Detection

In the system, visual machines are equipped with an Intel Core i5-5350U CPU 1.8 GHz processor with 2-GB RAM, and the host computer is equipped with an Intel Core i5-4590 3.3 GHz processor with 4-GB RAM. The experimental results are evaluated by two sub-tasks. One is to evaluate how fast the image processing is (efficiency), and the other is to assess the capability of the algorithm in detecting defect images (accuracy). For the sake of testing the speed of the system, in the experiment, 2400 magnetic rings are continuously detected and the execution time of each vision machine is calculated. As shown in Table 1, for different vision machines, it can be found that the maximum detection time of a single magnetic ring is 510 ms, and the maximum average detection time is 455 ms. In other words, the proposed detection system eventually reaches an average detection speed of about 120 pcs/min.

For the second sub-task, in order to test the system accuracy for various defects’ detection, we select 200 magnetic rings for each type of defect and test them. Table 2 demonstrates the statistical results. By counting the results of various defects’ detection, it can be concluded that the accuracy for crack and large-area defects, such as adhesion, hanger adhesion and pitting, reaches 100%. Meanwhile, the accuracy for foreign matters and fibers achieves 92.5% and 91.5%, respectively. From the results, it is confirmed that the proposed method and system possess good performance. Both the accuracy and the speed are suitable for magnetic ring real-time online inspection in the automated production line.

## 5. Conclusions

In this paper, we propose a defect detection system for magnetic rings and adopt advanced multi-camera vision technology. The system includes two efficient image processing algorithms, which are IERA and MRLA. By using the detection method for cracks, fibers and large-area defects based on connected domain filtering, it solves the difficult defect detection problem for three-dimensional magnetic rings with high speed and precision. The experiment results demonstrate that machine vision can substitute most of the manual operations in magnetic ring defect detection, greatly improving the production efficiency and the automation degree.

In the developed system, the hardware platform and software system are studied and designed in depth, and the final developed system is experimentally verified. Compared with existing systems, the current system has more comprehensive functions and a good integration degree. The device and method have a high detection precision, can realize real-time online detection of three-dimensional magnetic rings, and can be widely applied in magnetic ring production.

## Figures and Tables

**Table 1:** Inspection time statistics of the vision machine.

Number of Visual Machines	Detected Content	Maximum Time Consumption (ms)	Minimum Time Consumption (ms)	Average Time Consumption (ms)
1	Outer and inner rings	510	395	436
2	Lower end face	470	412	425
3	Inner ring	260	180	220
4	Outer ring, inner ring	495	402	440
5	Outer ring, inner ring	490	406	443
6	Outer ring, upper end surface	500	420	455

**Table 2:** Defect detection statistics.

Number	Name	Total Number of Magnetic Rings	Number of Identifications	Recognition Rate
1	Crack	200	200	100%
2	Adhesion	200	200	100%
3	Hanger adhesion	200	200	100%
4	Pitting	200	200	100%
5	Foreign body	200	185	92.5%
6	Fiber	200	183	91.5%
